# Métastases génitales du cancer du sein: étude de 3 cas et revue de la littérature

**DOI:** 10.11604/pamj.2018.30.7.14839

**Published:** 2018-05-04

**Authors:** Olfa Zoukar, Anis Haddad, Amira Daldoul, Sonia Zaied, Amina Ben Salem, Ines Zouari, Raja Faleh

**Affiliations:** 1 Service de Gynécologie, Centre de Maternité et de Néonatologie de Monastir (CMNM), Tunisie; 2 Service de Carcinologie du Centre de Maternité et de Néonatologie de Monastir, Tunisie; 3 Service de Radiologie du Centre de Maternité et de Néonatologie de Monastir, Tunisie

**Keywords:** Cancer du sein, métastases, ovaires, Breast cancer, metastases, ovaries

## Abstract

Le cancer du sein est le cancer le plus fréquent chez la femme, et son pronostic dépend en grande partie de l'existence de métastases. Le foie, le squelette et les poumons en sont les sites les plus fréquents, alors que les métastases génitales semblent plus rares et moins connues. La découverte d'une masse ovarienne chez une femme présentant un antécédent de cancer du sein pose le problème de son origine primitive ou secondaire. La fréquence rapportée dans la littérature des métastases ovariennes est d'environ 20 à 30 %. Cependant, lorsque l'on découvre une masse ovarienne chez une femme atteinte de cancer du sein, il s'agit trois fois plus souvent d'une tumeur ovarienne primitive que d'une métastase. La localisation utérine cervicale ou corporéale est encore plus rare. Leur diagnostic est souvent tardif, en raison de leur latence clinique; l'échographie, notamment par voie endovaginale et couplée au Doppler couleur, et le Frottis cervico-utérin doivent être réalisés de première intention sachant que leur rendement pour leur dépistage des masses ovariennes semble très faible. L'augmentation des marqueurs tumoraux CA 15-3 et ACE doit conduire à la recherche de métastases, mais ne fournit pas d'orientation diagnostique vers un site métastatique précis. Enfin, c'est l'examen anatomopathologique qui permettra de poser le diagnostic avec certitude. Les auteurs rapportent 3 observations de métastases génitales de cancer primitif mammaire (deux ovariennes et une cervicale utérine) soulignent l'intérêt de l'examen génital précis et régulier dans la surveillance des cancers du sein et discutent les facteurs prédictifs de leur survenue.

## Abstract (en)

Breast cancer is the most common cancer among women. Prognosis depends, in large part, on the presence of metastases. Liver, skeleton and lungs are the most frequent metastatic sites, whereas genital metastases are more rare and less known. The detection of an ovarian mass in a woman with a history of breast cancer raises the question of its primary or secondary origin. The frequency of ovarian metastases reported in the literature is approximately 20-30%. However, when an ovarian mass is detected in a woman with breast cancer, primary ovarian tumor is diagnosed three times more often than a metastasis. Cervical, uterine or corporeal metastases are even more rare. They are often diagnosed late, due to their clinical latency; transvaginal ultrasound coupled with Color Doppler and Pap smear must be performed as first-line examinations knowing that their screening performance in patients with ovarian masses is deemed low. An increase in CA 15-3 and CEA tumor markers must lead clinicians to investigate for metastases, but it doesn’t provide diagnostic orientation toward a specific metastatic site. Finally, only anatomo-athological examination allows certain diagnosis. We here report 3 cases of genital metastases from primary breast cancer (two patients with ovarian metastases and one patient with cervico-uterine metastasis) in order to highlight the role of accurate and regular genital examination in the monitoring of patients with breast cancer and to discuss the predictive factors for their occurrence.

## Introduction

Le cancer du sein est le cancer le plus fréquent chez la femme, et son pronostic dépend en grande partie de l'existence de métastases. Le foie, le squelette et les poumons en sont les sites les plus fréquents, alors que les métastases ovariennes semblent plus rares et moins connues [1]. Leur diagnostic est souvent tardif, en raison de leur latence clinique; l'échographie, notamment par voie endovaginale et couplée au Doppler couleur, doit être réalisée de première intention. Enfin, c'est l'examen anatomopathologique qui permettra de poser le diagnostic avec certitude. La probabilité de survenue de lésions ovariennes secondaires est quatre à cinq fois plus élevée dans les carcinomes lobulaires infiltrants que dans les carcinomes canalaires infiltrants. La survie médiane est estimée à 2 ans, et la survie à 5 ans est d'environ 18 %.

## Méthodes

**Figure 1:**
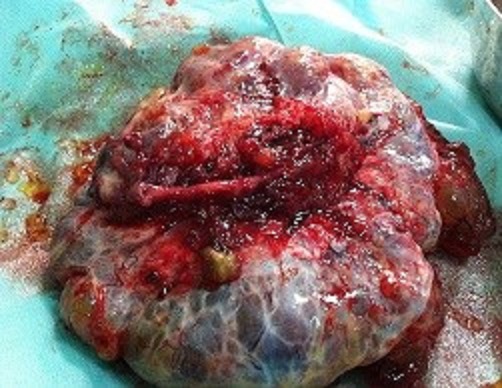
Métastase ovarienne d’un carcinome canalaire infiltrant du sein

**Observation 1:** Mme AA, âgée de 45 ans G8P5A3, non ménopausée, connue porteuse d'un carcinome mammaire gauche classée T4NOM1 (CCI, SBRI RH+ métastase osseuse diffuse), ayant nécessité 25 séances de radiothérapie, 12 cure de chimiothérapie et de l'hormonothérapie à base de tamoxifène. Une année après, l'échographie abdominale a objectivé une ascite de moyenne abondance avec présence de deux masses rétro utérine de 10 cm et de 9 cm chacune cloisonnée évoquant une tumeur ovarienne. Un complément par une imagerie par résonance magnétique (IRM) pelvienne vient d'affirmer le diagnostic. Le CA 125 est à 320 UI. La patiente a eu une hystérectomie totale avec annexectomie bilatérale. A l'examen anatomopathologiste : métastase ovarienne bilatérale d'un carcinome canalaire infiltrant mammaire. Il s'agit d'une tumeur mucineuse. (Figure 1). Le traitement a été complété par une reprise de la chimiothérapie. L'évolution était sans récidive après un an de traitement.

**Observation 2:** Une patiente âgée de 56 ans, ménopausée, ne présentant aucun antécédent médicochirurgical, nous a été adressée du service de gastrologie pour un complément d'exploration d'une ascite. L'échographie gynécologique a mis en évidence une masse annexielle hétérogène para-utérine, de consistance charnue, associée à une ascite abdominale. L'IRM pelvienne a confirmé la présence d'une masse para-utérine gauche faisant environ 90 x 87 mm de grand axe. Le dosage sanguin du CA 125 était élevé à 745 U/ml. Le frottis cervico-utérin était normal. Le dernier bilan sénologique de la patiente était strictement normal et datait d'un an. Devant ce tableau clinique et l'aspect de l'imagerie, une tumeur ovarienne gauche maligne a été fortement suspectée et la décision d'une laparotomie exploratrice a été retenue lors de réunion de concertation pluridisciplinaire. L'exploration peropératoire a montré une ascite de grande abondance, plusieurs petits implants péritonéaux, et un ovaire gauche d'aspect macroscopique malin. Nous avons pratiqué une hystérectomie totale avec annexcectomie bilatérale associée à une omentectomie et la résection des nodules tumoraux péritonéaux. L'analyse anatomopathologique de la pièce opératoire a montré sur l'ensemble des prélèvements une histologie et un profil immunohistochimique en faveur d'un adénocarcinome lobulaire mammaire. Une écho mammographie et une IRM ont été demande par la suite ce qui a permis de mettre en évidence la présence d'une lésion e 12mm située au niveau du quadrant inféro-interne du sein droit. La biopsie de cette lésion a conclu à un adénocarcinome lobulaire infiltrant de profil immunohistochimique semblable à la lésion ovarienne. Le dosage du CA 15,3 était élevé. Le bilan d'extension métastatique comprenant une scintigraphie osseuse et un scanner thoracoabdominal a été négatif. La patiente a bénéficié d'une chimiothérapie suivie d'une hormonothérapie par tamoxifène. Le bilan de surveillance post chimiothérapie les marqueurs tumoraux (CA15,3 et CA125) et le scanner thoraco- abdomino-pelvien, était normal. La patiente a également bénéficié d'un traitement conservateur au niveau du sein droit. L'analyse anatomopathologique de la pièce opératoire a montré un carcinome lobulaire infiltrant dont l'exérèse a été en marges saines.

**Figure 2:**
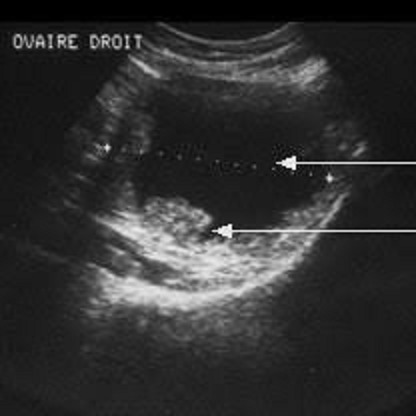
Échographique pelvienne : formation tissulaire ovarienne droite de 57 mm de grand axe avec des végétations endokystiques

**Observation 3:** Madame N.F. âgée de 63 ans, G4P3A1, ménopausée depuis 10 ans, diabétique sous ADO, ATCD de patey gauche il ya 4ans pour CCI sans métastase, découverte échographique lors du suivi annuelle habituel du cancer du sein, d'une ascite de moyenne abondance avec une formation tissulaire ovarienne droite de 57 mm de grande axe avec des végétations endokystique (Figure 2). Un complément d'IRM a confirmé l'aspect tissulaire végétant de la masse avec carcinose péritonéale et un ovaire gauche sain avec CA 125 à 320 UI. Une c'lioscopie diagnostique a été décidée et une annexectomie droite avec examen extemporané a été pratiquée. L'examen anatomopathologique extemporané a confirmé la nature maligne de la masse ovarienne droite, d'où la décision peropératoire d'une laparo-conversion avec une totalisation (hystérectomie totale avec annexectomie gauche) avec résection de quelques nodules de carcinose péritonéales et une omentectomie. L'analyse anatomopathologique de la pièce opératoire avait montré sur l'ensemble des prélèvements une histologie et un profil immunohistochimique en faveur d'un carcinome canalaire mammaire. Un staff multidisciplinaire fait de gynécologue, radiologue, anatomopathologiste et carcinologue a conclu à une chimiothérapie adjuvante. La surveillance pendant un an est sans récidive.

## Discussion

Le cancer du sein est le cancer le plus fréquent chez la femme, et son pronostic dépend en grande partie de l'existence de métastases. La découverte d'une masse ovarienne chez une femme présentant un antécédent de cancer du sein pose le problème de son origine primitive ou secondaire [1]. La fréquence rapportée dans la littérature des métastases ovariennes est d'environ 20 à 30 %, variable selon les études (autopsique, de castration thérapeutique ou clinique). Cependant, lorsque l'on découvre une masse ovarienne chez une femme atteinte de cancer du sein, il s'agit trois fois plus souvent d'une tumeur ovarienne primitive que d'une métastase. Leur diagnostic est souvent tardif, en raison de leur latence clinique; l'échographie, notamment par voie endovaginale et couplée au Doppler couleur, doit être réalisée de première intention mais son rendement pour le dépistage des masses ovariennes semble très faible [2]. L'augmentation des marqueurs tumoraux CA 15-3 et ACE doit conduire à la recherche de métastases, mais ne fournit pas d'orientation diagnostique vers un site métastatique précis. Enfin, c'est l'examen anatomopathologique qui permettra de poser (parfois non sans difficultés) le diagnostic avec certitude[3]. Les métastases ovariennes sont kystiques ou solides, sans prédominance nette d'une forme par rapport à l'autre ; il faut noter que l'atteinte ovarienne est microscopique dans un quart des cas. La probabilité de survenue de lésions ovariennes secondaires est quatre à cinq fois plus élevée dans les carcinomes lobulaires infiltrants que dans les carcinomes canalaires infiltrants. L'âge moyen lors du diagnostic de métastases ovariennes est compris entre 48 et 61 ans, et le délai moyen d'apparition de ces métastases est de 4 ans.

Le seul facteur pronostique retrouvé dans la littérature [4,5] est le volume tumoral résiduel après chirurgie, alors que le stade initial, le type histologique, le grade, le taux de récepteurs à l'oestradiol, le statut ménopausé et la bilatéralité de l'atteinte ovarienne semblent n'avoir aucune incidence sur le pronostic. La survie médiane est estimée à 2 ans, et la survie à 5 ans est d'environ 18 %. Trois cas de métastases mammaires à l'intérieur de tumeurs ovariennes primitives ont été décrits, respectivement dans un cystadénocarcinome mucineux, un fibrome et un thécome. La découverte de métastases ovariennes lors du diagnostic ou du suivi d'un cancer du sein apparaît donc comme un événement relativement fréquent, mais longtemps asymptomatique et peu recherché [6]. La prédominance du type lobulaire est clairement mise en évidence dans la littérature, mais la valeur pronostique de ces métastases ovariennes reste discutée, car elles sont fréquemment associées à d'autres localisations secondaires.

## Conclusion

Les métastases annexielles d'un cancer du sein sont souvent la manifestation d'une progression métastatique chez une patiente ayant un antécédent connu de cancer du sein mais peut plus rarement en être la manifestation initiale [7]. L'imagerie médicale est peu discriminante pour établir le diagnostic de masse annexielle métastatique [8]. En cas d'antécédent de cancer du sein, la laparoscopie semble être la voie d'exploration privilégiée pour établir le diagnostic histologique et évaluer la résécabilité des lésions [9]. Il semble que l'exérèse complète des métastases abdominales puisse offrir un bénéfice en termes de survie à ces patientes pour autant qu'il y ait eu un intervalle libre d'au moins cinq ans depuis le diagnostic initial de cancer du sein.

## Conflits d’intérêts

Les auteurs ne déclarent aucun conflit d'intérêts.
